# The Warren Shunt: Effect of Alcoholism on Portal Perfusion

**DOI:** 10.1155/1991/39364

**Published:** 1991

**Authors:** J. A. Myburgh

**Affiliations:** Department of Surgery University of the Witwatersrand Medical School York Road, Parktown Johannesburg 2193 South Africa


11. Niederhuber, J.E. (1985) Arterial chemotherapy for metastatic colorectal cancer in the liver. Conference Advantages in Regional Cancer Therapy. Giessen, West Germany
12. Weiss, L. (1989) Metastatic inefficiency and regional therapy for liver metastases from colorectal carcinoma. *Reg.Cancer Treat.*, **2**, 77–81
13. Albelda, S.M. and Buck, C.A. (1988) Integrins and other cell adhesion molecules. *FASEB J*, **4**, 2868–2880
14. Kanematsu, T., Matsumata, T., Takenaka, K., *et al.* (1988) Clinical management of recurrent hepatocellular carcinoma after primary resection. *Br.J.Surg.*, **75**, 203–206
15. Hughes, K.S., Sugarbaker, P.H. and other members of the Hepatic Metastases Registry. (1986) Resection of the liver for colorectal carcinoma metastases: A multi-institutional study of patterns of recurrence. *Surgery*, **100**, 278–284
16. Steel, G. Jr., Osteen, R.T., Wilson, R.E., Brooks, D.C., Mayer, R.J., Zamcheck, N. and Ravikumar, T.S. (1984) Patterns of failure after surgical cure of liver tumors. A change in the proximate cause of death and a need for effective systemic adjuvant therapy. *Am.J.Surg.*, **147**, 554–559
17. Bozzetti, F., Doci, R., Bignami, P. and Genarri, L. (1987) Patterns of failure following surgical resection of colorectal cancer liver metastases: rationale for a multimodal approach. *Ann.Surg.*, **205**, 264–270
18. Ekberg, J., Tranberg, K.G., Andersson, R., *et al.* (1987) Pattern of recurrence in liver resection for colorectal secondaries. *World J.Surg.*, **11**, 541–547
19. Kern, K.A., Pass, H.I. and Roth, J.A. (1987) Surgical treatment of pulmonary metastases. (In) Rosenberg SA. *Surgical Treatment of Metastatic Cancer*, pp 69–100. J.B.Lippincott Company: Philadelphia
20. Sugarbaker, P.H. (1991) Cytoreductive approach to peritoneal carcinomatosis: peritonectomy and intraperitoneal chemotherapy. *Post Advances in Colorectal Surgery*, **11-X**, 1–14
21. Consensus Statement. (1990) Adjuvant therapy for patients with colon and rectum cancer. NIH Consensus Development Conference, April 16–18, 1990, Vol.8 No.4
22. Hodgson, W.J.B., Friedman, M., Ahmed, T., *et al.* (1986) Treatment of colorectal hepatic metastases by intrahepatic chemotherapy alone or as an adjuvant to complete or partial removal of metastatic disease. *Ann.Surg.*, **203**, 420–425
23. Sugarbaker, P.H., Gianole, F.J., Dwyer, A.J. and Neuman, R. (1987) A simplified plan for follow-up of patients with colon and rectal cancer supported by prospective studies of laboratory and radiologic test results. *Surgery*, **102**, 79–83

Paul H Sugarbaker
Medical Director
The Cancer Institute
Washington Hospital Center
110 Irving Street, NW
Washington DC 20010-2975
United States of America

### THE WARREN SHUNT: EFFECT OF ALCOHOLISM ON PORTAL PERFUSION

### ABSTRACT

*Kawasaki, S., Henderson, J.M., Hertzler, G. and Galloway, J.R. (1991) The role of continued drinking in loss of portal perfusion after distal splenorenal shunt. Gastroenterology;* **100**, 799-804

**Fifty percent of patients with alcoholic cirrhosis who undergo distal splenorenal shunting for variceal bleeding lose portal perfusion within 1 year. Although it was previously considered that this loss of portal flow was irrevocable, the present study shows that with resolution of alcoholic hepatitis, portal perfusion can be restored. A 34-year-old patient with alcoholic liver disease and a distal splenorenal shunt lost portal perfusion 1 year after the operation. He had continued to drink alcohol and had high sinusoidal pressure. Following forced abstinence over the next 2 years, his sinusoidal pressure fell, liver colume decreased, results of liver biopsy improved, and portal perfusion was restored. Shunt patency was documented, and the same collaterals from the portal vein to the shunt could still be visualized as had been seen when portal flow was absent. Restoration of portal perfusion was attributed to decreased intrahepatic resistance secondary to abstinence from alcohol. A return to drinking in the next 9 months led to alcoholic hepatitis and once again loss of portal perfusion. This study places emphasis on increased intrahepatic resistance rather than the development of portal-to-shunt collaterals as important in the loss of portal flow in such patients.**

### PAPER DISCUSSION

KEY WORDS: Portacaval shunt, distal splenorenal shunt, portal hypertension, alcoholic liver disease

There is little dispute about the ability of the distal splenorenal shunt (DSRS) to prevent recurrent gastro-oesophageal bleeding from varices and portal hypertensive gastropathy[1-4]. This is achieved with a low incidence of postshunt encephalopathy. However, the conceptual validity of maintained *selective* gastro-oesophageal decompression has been challenged[5,6]. This is particularly so in the case of DSRS in patients with alcoholic cirrhosis, with reported loss of prograde portal venous perfusion in 50% to 70% of these patients[7,8].

In general persistence of prograde portal venous perfusion after DSRS depends on a favourable ratio between intrahepatic resistance and portomesenteric-gastrosplenic collateral (PM-GS) resistance. Maintained portomesenteric venous hypertension after DSRS provides the stimulus for the inexorable further development of potentially hepatofugal collaterals. Technical details of the operation are important in the prevention of loss of prograde portal venous perfusion. Inokuchi *et al.*[9] and Warren *et al.*[10] reported the potential importance of transpancreatic collaterals, the "pancreatic siphon", and the Emory group have reported the beneficial effects of complete splenopancreatic disconnection (SPD) in preventing loss of prograde portal venous perfusion in alcoholic cirrhotics[11].

The importance of gastro-spleno-colic disconnection to prevent the development of a potentially massive hepatofugal epiploic to shunt portal venous "steal" has been emphasized[2], and left gastric (coronary) veins may be multiple and must be sought and divided assiduously. Postoperative portal vein thrombosis is another cause of loss of prograde portal venous perfusion[2,3] but its occurrence is not frequent and has not been correlated clearly with the method of dealing with the stump of the splenic vein. Two other major identifiable PM-GS collateral pathways are transgastric and retroperitoneal, but these are not addressable by technical manoeuvres.

In the paper under review the Emory group have provided compelling evidence, on the basis of one carefully studied patient, for another important factor in the loss of prograde portal venous perfusion in patients with alcoholic liver disease undergoing DSRS. They have clearly documented the loss of prograde portal venous perfusion due to the increased sinusoidal resistance associated with acute alcoholic hepatopathy, the reversal of this loss following resolution of acute hepatopathy during unequivocal abstinence from alcohol, and the reappearance of hepatofugal flow with resumption of alcohol abuse. The authors state that this case is representative of patients who have reversible liver disease, but that these observations are probably not applicable to patients with stable or advanced cirrhosis.

My experience with DSRS in 141 patients, 76 having established alcoholic cirrhosis, suggests that the latter statement is not necessarily correct[3,4]. Superimposed acute alcoholic hepatitis was present on admission in the majority of these patients. Patients were kept in hospital before shunting for 1 to 24 weeks, mean 6 weeks, until maximal clinical and biochemical improvement had occurred and liver biopsies showed resolution of acute alcoholic hepatitis. On admission 50% of these patients were Childs grade C and 40% were Childs grade B. At operation, after due preparation, only 1 patient remained in Childs category C and 57% had improved to Childs grade A.

Serial longitudinal studies of hepatic arterial and portal venous perfusion were performed up to 10 years after DSRS in 63 patients, using dimethyltriamine pentaacetic acid (DTPA) flow scintigraphy. Although failure to demonstrate prograde portal venous perfusion was seen only in patients with alcoholic cirrhosis, it was not as frequent as reported elsewhere (23%), it did not correlate with the degree of collateralization or the occurrence of portosystemic encephalopathy, and it was not progressive with time following operation. Furthermore there was no statistically significant effect of splenopancreatic disconnection, on either the incidence of loss of prograde portal perfusion, or on the absolute values of the T3 indices in 22 alcoholics and 22 non-alcoholics studied at different times after operation.

The policy of adequate, and if necessary, prolonged in-hospital preoperative preparation in this series is probably unique. It is felt that this policy, and regular and personal postoperative surveillance, have played a major role in the remarkable degree of durable postoperative abstinence from alcohol seen in the series. This in turn may have contributed largely to the differences observed between the DTPA portal venous perfusion data and the angiographic perfusion data reported in other series. The findings in the remarkable case study reviewed here lend further credence to this postulate.

### REFERENCES

1. Warren, W.D., Zeppa, R. and Forman, J. (1967) Selective transplenic decompression of gastroesophageal varices by distal splenorenal shunt. *Ann. Surg.*, **166**, 437–455
2. Warren, W.D., Millikan, W.J., Henderson, J.M., Wright, L., Kutner, M., Smith, R.B., Fulenwider, J.T., Salam, A.A. and Galambos, J. (1982) Ten years portal hypertensive surgery at Emory. *Ann. Surg.*, **195**, 530–542
3. Myburgh, J.A. (1987) Selective shunting procedures in portal hypertension: Current perspectives. In: Nyhus, L.M., Ed. *Surgery Annual.* Norwalk, Connecticut. Appleton & Lange. pp. 83–109

4. Myburgh, J.A. (1990) Selective shunts: the Johannesburg experience. *Amer. J. Surg.*, **160**, 67–74
5. Maillard, J.N., Flamant, Y.M., Hay, J.M. and Chandler, J.G. (1979) Selectivity of the distal splenorenal shunt. *Surgery*, **86**, 663–670
6. Belghiti, J., Grenier, M.D., Nouel, O., Nahum, H. and Fekete, F. (1981) Long-term loss of Warren's shunt selectivity: Angiographic demonstration. *Arch. Surg.*, **116**, 1121–1124
7. Rikkers, L.F., Rudman, D., Galambos, J.T., Fulenwider, J.T., Millikan, W.J., Kutner, M., Smith, R.B., Salam, A.A., Jones, P.J. and Warren, W.D. (1978) A randomized controlled trial of the distal splenorenal shunt. *Ann. Surg.*, **188**, 271–282
8. Henderson, J.M., Millikan, W.J., Wright-Bacon, L., Kutner, M.H. and Warren, D.W. (1983) Hemodynamic differences between alcoholic and non-alcoholic cirrhosis following distal splenorenal shunt - Effect on Survival? *Ann. Surg.*, **198**, 325–334
9. Inokuchi, K., Beppu, K., Koyanagi, N., Nagamine, K., Hashizume, M. and Sugimachi, K. (1984) Exclusion of non-isolated splenic vein in distal splenorenal shunt for prevention of portal hypertension. *Ann. Surg.*, **200**, 711–717
10. Warren, D.W., Millikan, W.J., Henderson, K.M., Abu-Elmagd, K.M., Galloway, J.R., Shires, G.T., Richards, W.O., Salam, A.A. and Kutner, M.H. (1986) Splenopancreatic disconnection: Improved selectivity of distal splenorenal shunt. *Ann. Surg.*, **204**, 346–355
11. Henderson, J.M., Warren, W.D., Millikan, W.J., Galloway, J.R., Kawasaki, S. and Kutner, M.H. (1989) Distal splenorenal shunt with splenorenal disconnection: A 4 year assessment. *Ann. Surg.*, **210**, 223–341

J.A. Myburgh
Department of Surgery
University of the Witwatersrand
Medical School
York Road, Parktown
Johannesburg, 2193
South Africa

### SHOULD LAPAROSCOPIC SURGERY BE AN OUTPATIENT PROCEDURE?

### ABSTRACT

*Reddick, E.J. and Olsen, D.O. (1990) Outpatient laparoscopic laser cholecystectomy. The American Journal of Surgery,* **160**, 485-489.

**Laparoscopic laser cholecystectomy has been performed clinically in the United States since 1988. After refinement of the technique, the procedure was offered on an outpatient basis. Eighty-three patients underwent laparoscopic laser cholecystectomy during the study period. Thirty-seven (45%) had the procedure as an outpatient. Younger patients were more suited for the outpatient procedure and those without previous surgery were more likely to have the procedure done as an outpatient. Weight, operating time, and gallbladder pathology were similar, although patients with acute inflammation of the gallbladder were more likely to require hospitalization. The primary reason for patient admission was patient preference.**

